# Errors in Figure 3

**DOI:** 10.1001/jamahealthforum.2021.4958

**Published:** 2022-01-14

In the article titled “Assessment of State and Federal Health Policies for Opioid Use Disorder Treatment During the COVID-19 Pandemic and Beyond”^1^ published online on November 19, 2021, there were errors in Figure 3 regarding the reporting on Arizona, Arkansas, and Nevada. This article was corrected online.
